# Boundary Management Permeability and Relationship Satisfaction in Dual-Earner Couples: The Asymmetrical Gender Effect

**DOI:** 10.3389/fpsyg.2018.01723

**Published:** 2018-09-13

**Authors:** Marcello Russo, Ariane Ollier-Malaterre, Ellen Ernst Kossek, Marc Ohana

**Affiliations:** ^1^Bologna Business School, University of Bologna, Bologna, Italy; ^2^Department of Management, Kedge Business School, Bordeaux, France; ^3^ESG School of Management, Université du Québec A Montréal, Montreal, QC, Canada; ^4^Krannert School of Management and Susan B. Bulter Center for Leadership Excellence, Purdue University, West Lafayette, IN, United States

**Keywords:** boundary management, dual-earner couples, gender, relationship satisfaction, partner agreement

## Abstract

Given the increasing use of technology and the growing blurring of the boundaries between the work and nonwork domains, decisions about when to interrupt work for family and vice versa can have critical implications for relationship satisfaction within dual-earner couples. Using a sample of 104 dual-earner couples wherein one of the partners is a member of the largest Italian smartphone-user community, this study examines how variation in boundary management permeability within dual-earner couples relates to partner relationship satisfaction, and whether the effect differed by gender and partners’ agreement on caregiving roles in the family. Using actor–partner analysis, we examined the degree to which an individual and his or her partner’s level of family-interrupting work behaviors (FIWB, e.g., taking a call from the partner while at work) and work-interrupting family behaviors (WIFB, e.g., checking work emails during family dinner) was positively related to relationship satisfaction. Results show that women experienced greater relationship satisfaction than men when their partners engaged in higher levels of FIWB, and this relationship was stronger when partners had perceptual congruence on who is primarily responsible for caregiving arrangements in the family. This study advances research on dual-earner couples by showing the importance of examining boundary management permeability as a family social phenomenon capturing transforming gender roles.

## Introduction

Contemporary dual-earner couples face different challenges in managing work and nonwork relationships than did prior generations, when most men worked as the primary breadwinner, and women stayed at home to manage caregiving (Kanter, 1977). The multitude of everyday work, family, and personal issues to handle (Nomaguchi and Milkie, 2015) takes place in a social context where proliferating personal communication devices, such as cell phones, are likely to blur work–home boundaries (Olson-Buchanan and Boswell, 2006). These trends can make work–family issues increasingly challenging to navigate, with implications for couples’ relationships and negotiating gender roles. As an illustration of this, if one partner is always expected to interrupt work so as to handle family issues, it is likely that this situation will have a negative impact not only on career but also on relationship satisfaction, particularly if the partner feels that the other should be more involved in caregiving responsibilities. Not wanting (or feeling able) to sacrifice work time for family, some couples have renounced parenthood (Friedman, 2013) or opted out of dual careers (Radcliffe and Cassell, 2014). Thus, a growing challenge for contemporary dual-earner couples is how to build a system of boundaries whose permeability, which refers to the ease with which individuals situated in one role manage tasks related to another role (Ashforth et al., 2000), matches each other’s personal preferences and needs (Kossek and Lautsch, 2012; Lanaj et al., 2014; Dumas and Sanchez-Burks, 2015).

Although scholars suggest that it is important to take into account stakeholders’ boundary management preferences in addition to the focal individual’s (Kreiner et al., 2009), most prior research has adopted an individual level of analysis, focusing only on individual boundary management styles (Bulger et al., 2007; Kossek and Lautsch, 2008), defined as the tactics individuals use to secure their preferred level of permeability in alignment with their personal work and family identities (Kossek et al., 2012). Relatively little scholarly attention has been given to how congruence in boundary management styles affects a partner’s relationship satisfaction and variation in gender differences (Allen et al., 2014; Rothbard and Ollier-Malaterre, 2015).

Trefalt (2013) examined the issue of boundary management in dyadic relationships, focusing on how attorneys engaged in different boundary management decisions (avoidance vs. approach boundary setting) according to the nature of the relationship with potential violators, such as colleagues or clients. However, Trefalt’s study was limited to workplace relationships. In the current study, we aim to extend this stream of research to dual-earner couples via the Actor–Partner Interdependence Model (APIM, Garcia et al., 2015) to understand how variation in partners’ boundary management permeability relates to relationship satisfaction. Such research is important, as individuals are embedded in a multitude of roles beyond the workplace (Mitchell et al., 2001), and their partners shape many of their daily work and nonwork attitudes and behaviors (Carlson et al., 2015).

Boundary (Ashforth et al., 2000) and border (Clark, 2000) theories suggest that individuals’ creation and maintenance of boundaries vary along a continuum ranging from complete segmentation to complete integration of domains (Rothbard et al., 2005; Dumas and Sanchez-Burks, 2015). Kossek et al. (2012) recently argued that it is crucial to consider cross-role interruptions, i.e., the “intrusions from one role to another” (Allen et al., 2014, p. 109), because individuals may not always have control over their boundaries, and thus their enacted behaviors may differ from their preferences. In this endeavor, we focus on cross-role interruptions, as an indicator of the actual enactment of permeability couples’ work and nonwork boundaries. Because boundaries may be strong when protecting one role and weak when protecting another (Clark, 2000; Kossek et al., 2012), we focus on work-interrupting-family-behaviors (WIFB) (i.e., enabling work distractions to interfere with the family but not vice versa) as well as on family-interrupting-work-behaviors (FIWB) (i.e., enabling distractions from the family domain to interfere with the work but not the opposite).

**FIGURE 1:**
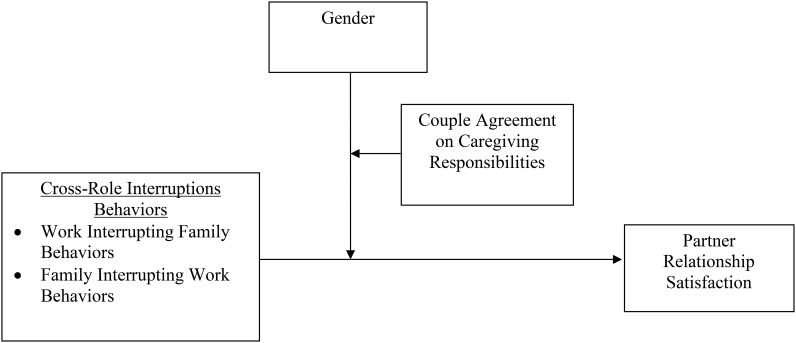
The impact of cross-role interruptions behaviors on partner relationship satisfaction as moderated by gender and couples’ agreement on caregiving responsibility enactment.

Drawing on crossover (Westman, 2001; Westman and Etzion, 2005) and gender role (Wood and Eagly, 2015) literatures, we examine whether coupled individuals’ cross-role interruptions influence their relationship satisfaction. Moreover, because how partners manage work and nonwork boundaries may be influenced by gender roles, as women have traditionally been the primary caregivers in the family (as illustrated in **Figure 1**), we also examine whether there are significant gender differences in this relationship and whether partners’ congruence in perceptions of who is the primary manager of caregiving responsibilities in the family has an impact on the strength of this relationship. To test our model, we selected a group of 104 dual-earner couples, wherein one of the partners was a member of the largest Italian smartphone-user community. This population is particularly interesting, as it enables us to study the phenomenon of increasing boundary blurring technologies in a country in which traditional gender role norms prevail (Riva, 2016). Indeed, Italy has one of the highest smartphone penetrations in the world (Newzoo, 2017) but still its prototypical couples still comprise male breadwinners and female homemakers (Craig and Mullan, 2010; Dotti Sani, 2014). Thus, examining what consequences the boundary management decisions aimed at regulating work and family interruptions generate on coupled men and women’s relationship satisfaction can help to identify potential gender differences associated with the increasing use of communication technologies, which is the cause of frequent episodes of role blurring (Olson-Buchanan and Boswell, 2006).

This paper advances research on boundary management in three important ways. First, this study extends prior research by shedding light on the undertheorized fit between the enacted boundary management behaviors and the partners’ relationship satisfaction. We extend Trefalt (2013) work on the impact of dyadic workplace relationships on boundary management effectiveness to the family sphere. Second, this study contributes to the literature on dual-earner couples (Becker and Moen, 1999; Solomon and Jackson, 2014; Ferguson et al., 2016) by testing if the degree of couple agreement on caregiving responsibilities is an important condition that can determine the success (or failure) of boundary management behaviors, a phenomenon that is pivotal in contemporary work–family research (Bianchi and Milkie, 2010). Third, we explore how boundary management permeability may capture transforming gender roles, examining the positive effects on women’s relationship satisfaction when men engage in high family to work-interruption behaviors.

## Theoretical Foundation

Individuals are embedded in a larger socio-emotional unit, namely, the family, which can exert a great influence on thoughts, emotions, and behaviors (Hammer et al., 2003, 2005). Ollier-Malaterre et al. (2015) suggest that an individual’s ability to maintain his or her work–life balance in the midst of a macro life transition, such as relocation to another country, divorce, or arrival of a baby, may depend on interactions, negotiations, and problem-solving with significant work and family stakeholders, including the partner. Thus, it is likely that an individual’s cross-role interruptions, and specifically his or her engagement in WIFB and/or FIWB, can influence the partner’s attitudes and behaviors in the home domain.

Crossover and spillover research also suggest that the demands associated with one partner’s work may interfere with the functioning and emotions experienced in the family domain (Matthews et al., 2006; Halbesleben et al., 2012). Piotrkowski et al. (1987) have been among the first scholars to demonstrate that an individual’s emotions and work-related stress spill over into the private domain, thus influencing the quality of marital relationships. Similarly, Doumas et al. (2003, 2008) have demonstrated that spillover is a relevant phenomenon both in single- and dual-earner couples, affecting the quality of marital relationship. While spillover focuses on cross-domain interference of one’s emotions within the same person, crossover focuses on influences across persons, as it examines how an individual’s role experience influences emotions, stress, and behaviors of other people in the same social environment. Crossover scholars (e.g., Westman and Vinokur, 1998; Westman, 2001) have demonstrated that the transmission of one’s role experiences to one’s partner can be direct when partners become empathetic of each other’s affective states, e.g., spurious when partners share some common concerns (e.g., financial problems) that lead them to experience similar affective states, or indirect, when the demands associated with one’s role affect the partner by reducing the communication quality and the participation in the other role, resulting in less mutual support (Westman, 2001). Based on this line of reasoning, we hypothesize that, when the focal actor engages in WIFB, such as when he or she responds to a work-related call during a family meal or completes work during time spent at home, the partner’s relationship satisfaction can be negatively affected, as the focal actor’s work-related interruptions can diminish the quality of the time spent together as well as the participation of the focal actor into the family. Conversely, we contend that engaging in FIWB, such as responding to a call from school while being at work or taking care of an urgent family matter during working hours, can be perceived by the partner as a sign of support and engagement with the family, with positive repercussions on relationship satisfaction. Thus, we hypothesize the following:

Hypothesis 1: Individuals’ WIFB are negatively related to their partner’s relationship satisfaction.
Hypothesis 2: Individuals’ FIWB are positively related to their partner’s relationship satisfaction.

## The Moderating Role of Gender

We also hypothesize that the effects of a focal actor’s cross-role interruptions on the partner’s relationship satisfaction are moderated by gender. We draw on the gender role literature to explain this hypothesis. Gendered beliefs concerning men and women’s division of labor consist of a series of role-specific norms and expectations that instill among individuals the pressure to behave in a way that is consistent with prototypical masculine and feminine roles in society (Eagly and Wood, 1999). Gender norms vary across cultures. For example, Scandinavian countries have high gender egalitarianism (House et al., 2004), meaning that both men and women typically work outside the home, and that “masculine” and “feminine” roles cross genders easily. Other countries, like Italy, operate within a breadwinner-homemaker framework (Pfau-Effinger, 2000; Lewis, 2009). In such countries, women are expected to be the primary contact for dependent care and to handle most of the domestic chores, even if they hold professional roles (Dotti Sani, 2014). Instead, men are expected to devote most of their time to work activities to provide financial stability to their family, even if it means working long hours and being absent from home for most of the day/week. Gender norms can have significant implications on individuals’ boundary management permeability. For example, in Italy it is typically tolerated that men have permeable family boundaries and engage in WIFB (but not vice versa), whereas women are expected to have permeable work boundaries and engage in FIWB (but not vice versa) (Riva, 2016; Ollier-Malaterre, 2018).

Drawing on this logic, we argue that the strength of the relationship between a focal actor’s cross-role interruptions and the partner’s relationship satisfaction varies according to the gender of the enactor of cross-role behaviors. Specifically, we suggest that WIFB will be more strongly negatively related to the partner’s relationship satisfaction when it is the woman who engages in WIFB rather than the man, as women who engage in frequent work-related interruptions behave in a way that is inconsistent with traditional gender norms. This line of reasoning is supported by prior research showing that men and women experience higher emotional distress and relationship dissolution when their behaviors deviate from prevailing gender roles (West and Zimmerman, 1987; Faulkner et al., 2005), whereas they experience greater stamina when they enact roles in a gender-consistent manner (Vohs et al., 2005). Therefore, we hypothesize the following:

Hypothesis 3a: The relationship between an individual’s WIFB and the partner’s relationship satisfaction is moderated by gender such that women’s WIFB is more strongly negatively related to their partner’s relationship satisfaction than men’s WIFB.

Regarding family-related interruptions occurring at work, we contend that the relationship between a focal actor’s FIWB and the partner’s relationship satisfaction will be stronger and more positive when it is the man who engages in FIWB rather than woman. Because Italian women are expected to prioritize family over work (Riva, 2016; Ollier-Malaterre, 2018), the impact of women’s FIWB on men’s relationship satisfaction can be minimal because men may consider women’s interruptions to take care of the family as normal and necessary to fulfill the basic responsibilities and obligations specified in their role even if they are working. In contrast, men’s engagement in FIWB can be noteworthy, as it is a behavior that significantly diverts from traditional gender norms; thereby, it can be more impactful on women’s relationship satisfaction. Opportunities for both men and women to participate equally into the labor market are associated with greater relationship satisfaction for women (Komter et al., 2012; Keizer and Komter, 2015). Thus, we hypothesize the following:

Hypothesis 3b: The relationship between an individual’s FIWB and the partner’s relationship satisfaction is moderated by gender such that men’s FIWB is more strongly positively related to their partner’s relationship satisfaction than the women’s FIWB.

## Couple Agreement on Caregiving Responsibilities and Gender as Moderators of the Relationship Between Wifb and Relationship Satisfaction

We also consider the role of partners’ agreement on caregiving responsibilities as a critical moderator of the relationship between cross-role interruptions and relationship satisfaction. Prior research has shown that partners’ agreement is crucial when examining the consequences of work–family experiences within couples (i.e., Streich et al., 2008; Wilson et al., 2018), as partners live in the same social system influencing each other (Hammer et al., 2003, 2005). Regarding boundary management, albeit the concept of agreement has never been studied in prior research, several studies have shown that couples engage in consultation, bargaining, and agreement when deciding the level of permeability of their boundaries (Carlson et al., 2015; Ferguson et al., 2016). Given this previous research, we suggest that, when partners agree on who is primarily responsible for caregiving activities in the family, they will experience less negative consequences when engaging in cross-role interruptions. Moreover, we contend that the role of couple agreement is even more critical for women, as gender role expectations in the Italian society are more salient for women than for men, strongly influencing their decisions regarding the engagement in work and family activities. As an illustration, imagine a couple in which partners agree that that the male partner will be in charge of caregiving arrangements and the female partner will be more focused on the job. In such a couple, it is possible that women’s higher engagement in WIFB will have a lower impact on men’s relationship satisfaction than in couples wherein partners have not reached such agreement, as this behavior reflects a couple’s shared decision. Thus, we hypothesize the following:

Hypothesis 4: The relationship between an individual’s cross-role interruption behaviors and the partner’s relationship satisfaction is moderated by the couple’s agreement on caregiving responsibilities and the partner’s gender such that the actor’s engagement in WIFB will be less strongly negatively related to the partner’s relationship satisfaction when the couple agrees on the allocation of caregiving activities in the family, especially when the actor engaging in WIFB is the woman rather than the man.

## Materials and Methods

### Participants and Procedures

Participants in this study were members of one of the largest Italian communities of smartphone users^1^. This community accounts for more than 5000 members and registers more than 2.5 million unique visitors per month on its websites. In 2012, we contacted a web manager and asked for collaboration in recruiting potential participants. Three recruiting messages were posted on the home page of the community’s website at 3-week intervals with a link directing to an online registration form that specified the study’s requirements: (i) to be a full-time employee and (ii) to be engaged with a cohabiting partner who has a full- or part-time job. Three hundred and twenty respondents completed the survey. Among them, 33 respondents were excluded because they did not meet the inclusion requirements. The total number of usable surveys was 287. Respondents were asked to invite their partner to participate in the study. To increase partners’ participation, we set up a drawing lottery for five iTunes gift cards prize of 50€ each. One hundred and thirty-one participants encouraged their partners to participate in the study, which made a response rate of 41%. Partners were sent a separate link to access a questionnaire containing the study’s variables. This procedure was followed to prevent participants from taking the study twice, for themselves and the partner. Based on our selection criteria, 27 couples were excluded from the study because they were not in a dual-earning situation. The final sample consisted of 104 heterosexual couples. Sample characteristics are described in **Table 1**. Participating couples are quite illustrative of the Italian society, as men worked on average longer hours than women (39.71 vs. 33.51 h, *p* < 0.05), and women who were more educated than men but nevertheless hold less managerial roles in companies than their male counterparts (even if those differences are not significant). Also typical of Italian society, women were employed in great numbers in occupations typically dominated by women, such as healthcare and education, whereas men were employed in greater numbers in the high-tech, manufacturing, and finance industries (*p* < 0.05). Finally, participants were asked to indicate the number of children they had to care at home. Only 31 couples (30%) answered to this question; whereas 73 couples preferred not to answer. A possible reason for such high number of missing values may be that Italian parents could perceive this question to be intrusive given that they are often criticized for not encouraging their children to leave home even when they are adults and have a job. Among these 31 couples who answered the question, 42% had one or more children they had to care at home. Due to the high number of missing variables, we have decided not to include this variable in further analyses.

**Table 1:** Description of the demographic characteristics of the study’s sample.

	Men	Women
Average age	34.56	36.13
Organizational tenure	7.15	7.72
Relationship tenure	9.5	
Average number of working hours	39.5	33.5
Education		
Bachelor	44%	52%
High school	48%	40%
Job status		
Managers	16%	9%
Employees	60%	59%
Consultants	11%	19%
Internship	8%	11%
Self-employed	5%	2%
Job industry		
High-tech	24%	8%
Manufacturing	15%	8%
Education	10%	15%
Healthcare	9%	14%
Financial services	9%	6%
Public administration	8%	6%
Trade	4%	6%
Service and consulting	3%	9%
Arts and culture	3%	4%
Media	2%	3%
Others (e.g., utilities, etc.)	13%	21%

*Notes: Age, relationships, and organizational tenures are expressed in years. N = 104 couples.*

### Measures

#### Cross-Role Interruptions

Items for measuring cross-role interruptions were from Kossek et al. (2012) and measured the two directions of interruptions: WIFB (five items) and FIWB (five items). Participants were asked to indicate the extent to which they agreed with the proposed statements. Answers were collected using a 5-point Likert scale, ranging from 1 = strongly disagree to 5 = strongly agree. The item to measure work-interrupting-family-behaviors (WIFB) were as follows: “I regularly bring work home”; “I respond to work-related communications (e.g., emails, texts, and phone calls) during my personal time away from work”; “I allow work to interrupt me when I spend time with my family or friends”; “I usually work during my vacations”; “I usually bring work materials with me when I attend personal or family activities.” The item to measure work-interrupting-family-behaviors (FIWB) were as follows: “I take care of personal or family needs during work”; “I respond to personal communications (e.g., emails, texts, and phone calls) during work”; “I do not think about my family, friends, or personal interests while working so I can focus” (reverse-coded); “When I work from home, I handle personal or family responsibilities during work”; “I monitor personal-related communications (e.g., emails, texts, and phone calls) when I am working.” The Cronbach’s alphas were as follows: WIFB (0.85 for men; 0.77 for women) and FIWB (0.69 for men; 0.60 women).

#### Relationship Satisfaction

Relationship satisfaction was assessed with the five-item marital satisfaction scale by Norton (1983) by replacing the term “marriage” with “relationship” considering that not all the participating couples were engaged in a married relationship. Answers were collected using a 5-point Likert scale ranging from 1 = strongly disagree to 5 = strongly agree. A sample item is: “My relationship with my partner makes me very happy.” The Cronbach’s alphas were 0.88 for men and 0.91 for women.

#### Partners’ Agreement

To measure partners’ agreement on caregiving responsibilities, based on Kossek et al. (2001), we asked each partner to independently report who, in their opinion, was primarily responsible for caregiving arrangements in their family. Respondents could select two alternatives: (1) themselves and (2) the partner. Then, we coded the responses to create a dummy variable, with 1 indicating partners’ agreement and 0 indicating partners’ disagreement. We chose not to offer the option to answer that both partners were equally responsible for care, as this option might have induced socially desirable responses, especially among men, who like to consider themselves as egalitarian even if they enact nonegalitarian behaviors or prefer traditional home-centered partners (Keizer and Komter, 2015). Overall, 69% of couples agreed on who was primarily responsible for caregiving activities in the family and, again typical of Italy, in 79% of cases partners declared that the woman was most responsible for caregiving activities in the family; whereas the man was indicated as being mainly responsible for caregiving activities in only 21% of couples.

#### Demographic Covariate Measures

Following prior studies (Carlson et al., 2015; Ferguson et al., 2016), we included several demographic covariates in our analysis: *organizational tenure, relationship tenure, and number of hours worked per week.*

#### Couple Type Covariate Measure

The type of couple that partners form, which stems from their role identity, may influence the partners’ work–family decisions and behaviors, such as who is interrupting work for family and vice versa. Masterson and Hoobler (2015) identified five types of dual-earner couples: (1) traditional (i.e., a couple, wherein the man is mostly focused on work and the woman on family); (2) nontraditional (i.e., a couple, wherein the man is mostly focused on family and the woman on work); (3) family first (i.e., a couple, wherein both the man and the woman are more focused on family than work); (4) outsourced (i.e., a couple, wherein both the man and the woman are more focused on work than family, therefore outsourcing the activities of care); and (5) egalitarian (i.e., a couple, wherein the man and the woman are highly focused both on work and on family). Role identity is also considered by Kossek et al. (2012) to be an important dimension of an individual’s boundary management style. Therefore, we included measures of work and family identities and coded the partners’ responses so as to include couple type as a covariate. We used Kossek et al. (2006) to measure *work identity* (two items) and *family identity* (two items). Sample items are: “People see me as highly focused on my work” (work identity) and “I invest a large part of myself in my family life” (family identity). Two authors coded the couples’ typology based on the men and women’s score on the work and family role identity scales. We computed the difference for each partner between his or her work and family identity score and then assigned the couple to a couple scenario type. For example, when men reported higher work identity than family identity and women reported higher family identity than work identity, we coded such couples as being “traditional,” as suggested by Masterson and Hoobler (2015). If both the male and female partners had relatively equal and higher scores on both work and family identity scales, we coded the couple as “egalitarian.” We repeated this process using two coders and resolved disagreements by turning to Masterson and Hoobler’s definitions to make our final decision.

### Data Analysis

Data were analyzed with multilevel modeling using the APIM for distinguishable dyads with a between dyad’s moderator (Garcia et al., 2015). Such technique enabled us to test the relationships between a focal actor’s cross-role interruption and the partner’s relationship satisfaction while accounting for the nested structure of the data (individuals within couples). In this model, the “actor effect” represents the association between cross-role interruptions and relationship satisfaction *within a person*, namely, the effect of the actor A’s self-reported cross-role interruption and actor A’s self-reported relationship satisfaction. “Partner effect” represents the association *across people*, namely, the effect of actor A’s self-reported cross-role interruptions on partner B’s relationship satisfaction and vice versa. Relationship satisfaction was the dependent variable. Individual-level predictors, namely, self and partner reported cross-role interruptions, were explored at level 1, and couple-level variables, i.e., the moderator indicating the partners’ agreement on caregiving arrangements, were explored at level 2. Predictors were grand-mean centered before running the analyses. The analyses were conducted with HLM 7, using full maximum likelihood estimation and unstandardized standard errors.

## Results

**Table 2** displays the means and standard deviations for all variables, and **Table 3** shows the correlations between all the study’s variables, with men’s correlations above the diagonal and women’s correlations below the diagonal. We used paired samples *t* tests to examine gender differences on the study’s variables. No significant gender differences were found with regard to average levels of WIFB and relationship satisfaction. The difference between men and women’s FIWB was significant (mean difference = 0.17; *t* = 1.991; *p* < 0.5); surprisingly, men engaged in greater FIWB than women. Turning to dyadic analysis, interestingly, the within-dyad correlations were statistically significant, which supports the importance of examining relationships at the dyadic level using the APIM method, which accounts for nested nonindependent relationships.

**Table 2:** Means and standard deviations of the main study’s variables.

	Men		Women	
	M	S.D.	M	S.D.
Family-Interrupting-Work-Behaviors (FIWB)	3.38	0.73	3.20	0.72
Work-Interrupting-Family-Behaviors (WIFB)	2.93	0.96	2.77	0.86
Relationship satisfaction	4.22	0.72	4.25	0.78

*Notes: N = 104.*

**Table 3:** Correlation matrix for the study’s variables.

		i	ii	iii	iv	v	vi	vii
i	Relationship tenure	0.86^∗∗^	0.67^∗∗^	0.53^∗∗^	0.04	−0.09	0.05	−0.26^∗∗^
ii	Age	0.69^∗∗^	0.81^∗∗^	0.71^∗∗^	0.03	−0.09	0.12	−0.30^∗∗^
iii	Organizational tenure	0.59^∗∗^	0.76^∗∗^	0.58^∗∗^	0.07	−0.06	0.06	−0.22^∗^
iv	Hours worked per week	0.06	0.03	0.07	0.39^∗∗^	0.15	−0.16	0.12
v	FIWB	−0.23^∗^	−0.28^∗∗^	−0.19	0.09	0.24^∗^	0.26^∗∗^	0.25^∗^
vi	WIFB	0.01	0.01	0.01	−0.10	0.23^∗^	0.06	−0.07
vii	Relationship satisfaction	−0.06	−0.14	−0.11	0.18	0.03	−0.12	0.45^∗∗^

*^∗^p < 0.05, ^∗∗^p < 0.01. FIWB = Family-Interrupting-Work-Behaviors. WIFB = Work-Interrupting-Family-Behaviors. Correlations for men appear below the diagonal, whereas correlations for women appear above the diagonal. Correlations along the diagonal are between dyad members.*

The two first hypotheses examined the effects of the focal actor’s cross-role interruption behaviors, namely, WIFB and FIWB, on their partner’s relationship satisfaction. To test these hypotheses using the APIM, actor and partner effects were estimated in the same equation. Data from each dyad member were treated as nested scores within groups of two people. The predictor variables in the model included gender as well as actor and partner’s WIFB and FIWB. The hypotheses predicted a negative relation between individuals’ WIFB and their partner’s relationship satisfaction and a positive relation between individuals’ FIWB and their partner’s relationship satisfaction. As shown in **Table 4**, there was a significant negative association between WIFB and the perceived relationship satisfaction for the same individual (*b* = −0.12; *p* < 0.05) but no significant association between an individual’s WIFB and the partner’s level of relationship satisfaction (*b* = −0.01; *p* > 0.10). Thus, H1 was not supported, as results show that a partner’s relationship satisfaction was not significantly affected by the focal actor’s engagement in WIFB. H2 was supported by data, as the results indicate a significant and positive association between the actor’s FIWB and the partner’s relationship satisfaction (*b* = 0.17; *p* < 0.05).

**Table 4:** Results of HLM regression analysis of actor and partner effects of cross-role interrupting behaviors predicting relationship satisfaction.

	B	S.E.
Constant	4.23^∗∗^	0.06
Gender	0.001	0.04
FIWB actor	0.14^∗^	0.07
FIWB partner	0.17^∗^	0.07
WIFB actor	−0.01	0.56
WIFB partner	−0.12^∗^	0.56

*^∗^p < 0.05, ^∗∗^p < 0.01. FIWB = Family-Interrupting-Work-Behaviors. WIFB = Work-Interrupting-Family-Behaviors; Deviance = 431.97; d.f. = 8. Notes: B and S.E. indicate Standardized Beta coefficients with robust standard error.*

Hypotheses 3 and 4 proposed that the effect of an individual’s cross-role interruption behaviors on the partner’s relationship satisfaction differed for men and women and varied for couples, depending on the extent to which partners agreed or disagreed on caregiving responsibilities within the family. To test these hypotheses, “two-intercept” models were estimated (Kenny et al., 2006). In the first step, we introduced the dummies representing gender, the cross-role interruptions, and their interaction with each gender dummy. **Table 5** shows that the interaction term capturing the impact of women’s FIWB on their partner’s relationship satisfaction (FIWB partner × man) was positive and significant (*b* = 0.297; *p* < 0.01). In contrast, the interaction term capturing the impact of men’s FIWB on their partner’s relationship satisfaction (FIWB partner × woman) was not statistically significant (*b* = 0.05; *p* = 0.ns). In order to confirm the difference of attitudes between each couple regarding FIWB, we tested simple slopes using the HLM two–way interaction procedure recommended by Bauer et al. (2006).

**Table 5:** Results of double intercept models HLM regression analysis predicting relationship satisfaction.

		B	S.E.
Step 1	Man (intercept)	4.25^∗∗^	0.07
	Woman (intercept)	4.26^∗∗^	0.07
	FIWB Actor × Man	−0.01	0.10
	FIWB Partner × Man	0.29^∗∗^	0.10
	WIFB Actor × Man	−0.11	0.07
	WIFB Partner × Man	−0.02	0.08
	FIWB Actor × Woman	0.29^∗∗^	0.10
	FIWB Partner × Woman	0.05	0.10
	WIFB Actor × Woman	−0.12	0.08
	WIFB Partner × Woman	−0.03	0.07
Step 2	Man × Agreement	0.04	0.16
	Woman × Agreement	0.04	0.16
	FIWB Actor × Man × Agreement	−0.19	0.22
	FIWB Partner × Man × Agreement	0.02	0.29
	WIFB Actor × Man × Agreement	0.17	0.16
	WIFB Partner × Man × Agreement	0.19	0.18
	FIWB Actor × Woman × Agreement	0.32	0.29
	FIWB Partner × Woman × Agreement	−0.37^+^	0.22
	WIFB Actor × Woman × Agreement	0.20	0.18
	WIFB Partner × Woman × Agreement	−0.11	0.16

*^∗^p < 0.05, ^∗∗^p < 0.01, ^+^p < 0.10. FIWB = Family-Interrupting-Work-Behaviors. WIFB = Work-Interrupting-Family-Behaviors. Agreement = Agreement on caregiving roles. Notes: B and S.E. indicate Standardized Beta coefficients with robust standard error.*

**FIGURE 2:**
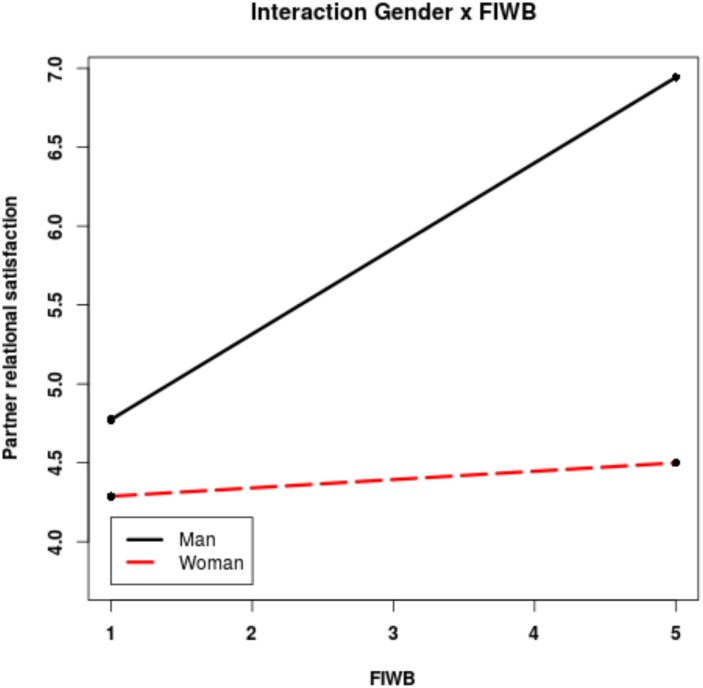
Results of interactions between gender and the degree to which individuals engaged in family interrupting work behaviors on partner’s relationship satisfaction.

We tested the significance of the slope concerning the moderating role of gender on the WIFB and FIWB and partner effects, i.e., whether the effect of engaging in higher levels of WIFB or FIWB on the partner’s relationship satisfaction was stronger for men or for women. H3a was not supported by data. No significant effects were found regarding an actor engaging in higher levels of WIFB and the partner effect and differences in patterns by gender. However, as shown in **Figure 2** and consistent with H3b, engaging in FIWB had a greater positive effect on the partner’s relationship satisfaction when men engaged in higher family-related interruptions at work than women. Results indicate that the simple slope was significant and positive only for men [simple slope = 0.5421(0.2364); *z* = 2.2928; *p* = 0.0219] but not for women [simple slope = 0.0528(0.1005); *z* = 0.5258; *p* = 0.599].

Hypotheses 4 was tested using a three–way interaction with gender, cross-role interruptions, and partners’ agreement on caregiving roles. We introduced in the models the interactions between partners’ agreement on caregiving roles, WIFB, and gender to analyze if partner agreement on caregiving responsibilities within the family strengthened or attenuated the relationship between cross-role interruption behaviors and relationship satisfaction for men and women. **Table 4** shows that the interaction between gender, WIFB and partner agreement was not significant (both *ps* > 0.25), failing to support H4. Notably, we also tested if our results differed among different types of couples based on Masterson and Hoobler (2015) couple typology, and we found no significant difference in our results, thereby suggesting that the different combination of men and women’s role identities did not significantly shape the relationship between an individual’s cross-role interruption behaviors and the partner’s relationship satisfaction^2^.

## Discussion

This study advances research on dual-earner couples by showing the importance of examining boundary management permeability as a family social–relational phenomenon capturing transforming gender roles. With the increasing use of cell phones and blurring work–life boundaries, couples must increasingly navigate work and nonwork interruptions throughout the day in ways that enable them to fulfill their work and family responsibilities. The goal of this paper was to examine the effects of individuals’ cross-role interrupting behaviors on the partner’s relationship satisfaction accounting for gender differences and partner agreement on the division of labor regarding caregiving responsibilities within the family. Drawing on prior research suggesting that couples’ work–family behaviors are highly influenced by gender norms (Nomaguchi and Milkie, 2015; Wood and Eagly, 2015), we examined whether individuals experienced higher or lower relationship satisfaction when their partner engaged in higher family interfering work behaviors (FIWB) or higher work interfering family behaviors (WIFB) and the extent to which gender and couples’ agreement on caregiving responsibilities played in influencing these relationships.

The results indicate that, across couples and for women especially, the extent to which an individual engages in higher or lower levels of FIWB, but not WIFB, can significantly shape a partner’s relationship satisfaction. The result of the two–way interaction analysis with gender reveals that women experienced higher levels of relationship satisfaction than men when their partner handled family issues while being at work, i.e., when they engaged in high FIWB. This result demonstrates that the examination of BM behaviors in dual-earner couples can be better understood by considering societal gender role expectations. Drawing on the gender role perspective, it is possible that the lack of significance between women’s engagement in FIWB and men’s relationship satisfaction is due to the belief that women who interrupt their work activities to take care of family issues are “simply adhering” to the basic societal expectations in traditional gender role countries like Italy (Riva, 2016; Ollier-Malaterre, 2018). Thus, engaging in FIWB may be unnoticed and less valued than in more egalitarian countries, thereby producing minimal effects on men’s relationship satisfaction. By contrast, it is possible that women are more satisfied of their relationship when men handle family-related activities at work, as this is an unexpected behavior that deviates from traditional gender norms, thereby becoming more noticeable and appreciated. Because prior research has found that contemporary women increasingly look for gender egalitarian partners (Press, 2004; Stanik et al., 2013), it is possible that being partnered with a man who is more involved in the family domain has positive repercussions on the relationship satisfaction for dual-earner women. Regarding the effects of couple agreement on the allocation of caregiving responsibilities, the results did not provide support to our hypotheses as neither women nor men experienced greater relationship satisfaction in presence of partners’ WIFB when the couple agreed on caregiving responsibilities.

### Theoretical Contributions

We contribute to the boundary management literature by extending emerging work on the relational nature of boundary management, which had focused on relationships in the workplace, to couples’ relationships. More specifically, our study extends prior research that emphasizes the relational context in which boundaries are crafted and negotiated (Trefalt, 2013); our findings indicate that not only workplace relationships matter but also dyadic intimate relationships in which boundary management decisions, such as how to manage caregiving responsibilities, are often discussed and negotiated. This is important because what happens in our romantic relationships can affect work as well (Turvey and Olson, 2006) and because these domains are increasingly connected (Hammer et al., 2003).

Our research thus extends the boundary management literature by emphasizing the value of focusing on couples as a unit (Luo and Klohnen, 2005) and by showing the importance of considering gender norms when analyzing the outcomes of boundary management. Our research extends and departs from prior research that examined the congruence between one’s personal preferences for segmentation/integration and stakeholders’ preferences at work or at home to ensure boundary management success (Kreiner et al., 2009). In the case of couples, we have demonstrated that it is also crucial to also consider the alignment with societal gender norms. This is important as, thus far, boundary management research has not acknowledged the influence of gender role norms on boundary management behaviors and success, whereas more studies examining gender roles have been conducted on work–family conflict and enrichment (Frone et al., 1992).

### Practical Implications

Our study has important practical implications for coupled individuals and organizations. Coupled individuals are rarely aware that their boundary management behaviors may affect the well-being of their partner’s as well as their own. Moreover, coupled individuals seldom understand that the way the couple manages the work and nonwork boundaries in accordance to pervasive gender norms, or in contrast with these norms, has consequences for their relationship satisfaction. For this reason, it may be worthwhile for couples to assess their partner’s boundary management preferences and behaviors and to examine how their family unit’s roles and responsibilities can best be met. This may imply explicit discussions about how career and family aspirations could be conciliated within the couple and how caregiving roles could be distributed in accordance with the legitimate preferences and aspirations of each partner.

As for organizational implications, our research should be of interest, as relationship tensions are among the major causes of distraction at work (Turvey and Olson, 2006), and they can have a significant impact on business operations and performance (Ferguson et al., 2012). It may be useful for organizations to include resources on healthy romantic relationships in corporate wellness and counseling programs with the goal of improving employees’ communication, conflict resolution, and parenting skills.

### Limitations and Future Research

The present study is not without limitations. First, causal directions cannot be interpreted due to the cross-sectional nature of data. However, the theoretical reasoning supporting our hypotheses is consistent with the literature on couples and marriage (e.g., Matthews et al., 2006; Stanik et al., 2013; Keizer and Komter, 2015), consistently reporting the effects of the division of household labor on marital relationships and not the other way around. For instance, Stanik et al. (2013) found that, in couples where the division of household labor reflected traditional gender role ideologies, women reported a lower level of love over time; whereas love remained stable over time when partners participated more equally in household labor. Likewise, Keizer and Komter (2015) found that, although men reported higher relationship satisfaction when they held more modern gender role attitudes, they reported significant lower relationship satisfaction when coupled with a female partner who did not adhere to traditional gender norms. Although the literature clearly supports causality in the direction we hypothesized, we recommend that future research uses longitudinal research designs that rule out the reverse causality. Such design would also account for changes individuals may experience in their boundary management behaviors across career and life stages (Rothbard and Ollier-Malaterre, 2015) and shed light on couples’ trade-off over their life course (Becker and Moen, 1999). From a methodological perspective, another limitation is the limited sample size with data collected in just one country, which reduced the generalizability of our conclusions. Further research should replicate our model with a larger number of couples from countries with different levels of gender egalitarianism in order to confirm the robustness of our results.

Our paper, which is one of the first to acknowledge the importance of considering gender norms in boundary management within couples, opens up other avenues for future research. In particular, we encourage scholars to examine other variables of interest for couples such as the specificities of the caregiving responsibilities (i.e., number of children, number of hours spent caring for children, as well as for elder parents or handicapped adults), and the partners’ agreement on the cross-role interruption behaviors themselves. Other variables of interest at the individual level would be segmentation and integration preferences. Importantly, albeit our findings could be generalized to other countries in which traditional gender role norms prevail – that is a sizable part of the world (Ollier-Malaterre, 2018) – future research on couples’ dynamics in different countries, in particular countries presenting greater variation in their internal level of gender equality and including couples with different work and family circumstances (e.g., single earner families, same-sex partnerships), would be valuable. Last, it could be fruitful to measure gender role orientation at the individual level to be attuned to the idea of tightness vs. looseness of national cultures (Tsui et al., 2007) and capture potential within-country heterogeneity in perceptions of gender norms (Ollier-Malaterre et al., 2013).

## Conclusion

This study provides empirical evidence that connecting research on boundary management permeability, couples’ dynamics, and gender norms provides a richer understanding of the effects of boundary management behaviors in dual-earner couples. Our results suggest that there exists no unique best way to manage boundaries in a couple within a traditional gender role context; rather trade-offs and collaboration seem necessary to assess what boundary management behaviors may be suitable for the couple in a particular cultural context. We hope that this study sparks interest into further analysis of these trade-offs and of their outcomes.

## Ethics Statement

This study was carried out in accordance with the recommendations of “Recommendations for Conducting Research with Human Beings, Research Committee Rouen Business School, France” with written informed consent from all subjects. All subjects gave written informed consent in accordance with the Declaration of Helsinki. The research protocol was approved by the Research Ethics Commission of the Rouen Business School (now Neoma), in France, which was the primary institution of two authors at the time of data collection.

## Author Contributions

All authors listed have made a substantial, direct and intellectual contribution to the work, and approved it for publication.

## Conflict of Interest Statement

The authors declare that the research was conducted in the absence of any commercial or financial relationships that could be construed as a potential conflict of interest.

## Footnotes

1 The research protocol was approved by the Research Ethics Commission of the Rouen Business School (now Neoma), in France, which was the primary institution of two authors at the time of data collection.

2 Results available from corresponding author upon request.
